# Detecting and reconstructing breakage-fusion-bridge cycles from long-read sequencing using BFBArchitect

**DOI:** 10.1093/bioinformatics/btag225

**Published:** 2026-07-07

**Authors:** Chaohui Li, Siavash Raeisi Dehkordi, Daniel Muliaditan, Ramanuj DasGupta, Jens Luebeck, Kaiyuan Zhu, Vineet Bafna

**Affiliations:** Department of Computer Science and Engineering, University of California San Diego, La Jolla, CA 92093, United States; Department of Computer Science and Engineering, University of California San Diego, La Jolla, CA 92093, United States; Genome Institute of Singapore (GIS), Agency for Science, Technology and Research (A*STAR), 138634, Singapore; CRUK Scotland Institute, Glasgow G61 1BD, United Kingdom; School of Cancer Sciences, University of Glasgow, Glasgow G61 1QH, United Kingdom; Department of Computer Science and Engineering, University of California San Diego, La Jolla, CA 92093, United States; Department of Computer Science and Engineering, University of California San Diego, La Jolla, CA 92093, United States; School of Computer Science, Shanghai Jiao Tong University, Shanghai 200240, China; Department of Computer Science and Engineering, University of California San Diego, La Jolla, CA 92093, United States; Halicioğlu Data Science Institute, University of California San Diego, La Jolla, CA 92093, United States

## Abstract

**Motivation:**

Focal oncogene amplification is a key driver of tumor progression. Remarkably, the increased pathology depends on the context—whether the amplification is extrachromosomal (ecDNA) or intrachromosomal. EcDNA amplifications promote heterogeneity, therapy resistance, and poor prognosis. Focal intrachromosomal amplifications often arise through breakage-fusion-bridge (BFB) cycles, which produce highly rearranged but stable chromosomes. Distinguishing BFB from ecDNA remains challenging due to overlapping genomic signatures. To address this, we present BFBArchitect, a computational method leveraging long-read Oxford Nanopore data to identify BFB sequences consistent with both copy number and structural variations.

**Results:**

We provide a novel combinatorial characterization of BFB, which naturally leads to an integer linear programming (ILP) optimization. The ILP optimization generates a BFB sequence that best explains experimentally observed copy numbers and foldback structural variants. We implement this idea in a tool called BFBArchitect, which achieves near-perfect accuracy in distinguishing BFB from non-BFB structures in extensive simulations as well as on 18 validated tumor samples. Moreover, it generates sequence-level BFB reconstructions that provide mechanistic insights into BFB formation, including repair mechanisms with template switching and other structural variants, and recapture of telomere for stabilization.

**Availability and implementation:**

BFBArchitect is available at https://github.com/AmpliconSuite/BFBArchitect.

## 1 Introduction

Focal copy number (CN) amplification of oncogene is considered an important marker of pathology (Davoli *et al.* 2017). Indeed, multiple cytogenetic (e.g. testing for amplification of *HER2, MDM2, CCND1* in various cancer types) and/or microarray and panel tests of CN amplifications are routinely used as clinical biomarkers. However, these tests do not adequately determine an important aspect of amplification: is the amplification extrachromosomal (ecDNA) or intrachromosomal? Multiple experiments with large patient cohorts in recent years have shown that the survival outcomes for patients worsen significantly when oncogenes are amplified as ecDNA (Kim *et al.* 2020, 2024, Chapman *et al.* 2023, Bailey *et al.* 2024). Importantly, the random segregation of ecDNA into daughter cell creates heterogeneity, and allows the cancer cell to evolve toward an optimal number of copies for a particular environment (Lange *et al.* 2022). Not surprisingly, ecDNAs mediate resistance to targeted therapy (Song *et al.* 2022) and chemotherapy (Bailey *et al.* 2024).

While important, ecDNA is not the only mechanism of focal oncogene amplification. First, ecDNA can agglomerate and reintegrate into the chromosome, often in a non-native location to form stable intrachromosomal amplifications, visible as homogeneously staining regions (HSRs) in metaphase spreads. Next, other completely independent mechanisms can generate a chromosomal focal amplification, with the most important mode being breakage-fusion-bridge cycles (BFBs) (McClintock 1939, 1941). BFB cycles start with a telomere crisis, which is stabilized by a bridge formation, usually between sister chromatids. To formalize this idea, consider a chromosomal arm denoted by genomic segments 〈c〉1234〈t〉, with segment 1 at the centromeric end and 4 at the telomeric end. A telomeric crisis removing segment 4 and bridge formation would lead to the dicentric chromosome $\langle c\rangle 123\bar{3}\bar{2}\bar{1}\langle c\rangle$, where $\bar{3}\bar{2}\bar{1}$ are inverted duplicated segments on the sister chromatid. Amazingly, the bridge persists beyond cytokinesis through the G1 phase of the next cycle, where it is resolved through multiple mechanisms (Maciejowski *et al.* 2020, Umbreit *et al.* 2020, Guérin and Marcand 2022). Unequal mitotic separation and breakage of the bridged chromosomes (“bridge-resolution”) create daughter cells with broken ends, one with an inverted duplication on one chromosome, and a deletion on the other. In the previous example, breakage between $\bar{2}$ and $\bar{1}$ leads to a genome $\langle c\rangle 123\bar{32}$. The broken ends generate increased replication stress, resulting in DNA instability, chromothripsis, and even ecDNA formation (Ly *et al.* 2019, Umbreit *et al.* 2020).

However, in some cases, the broken ends result in continued BFB cycles. As with ecDNA, the inverted duplication may increase oncogene copy numbers, providing a proliferative advantage and selection for cells with high copy-number chromosomes. Thus, a small number of BFB cycles can lead to a highly rearranged genome. For example,


$$\langle c\rangle 123\to \langle c\rangle 123\bar{32}\to \langle c\rangle 123\bar{32}2\to \langle c\rangle 123\bar{32}2\bar{2}23\bar{32}.$$

The process continues until a telomere is reacquired. The process of telomere reacquisition can occur through neo-telomere formation (Flint *et al.* 1994), or through an unbalanced translocation (Meltzer *et al.* 1993), but these have not been systematically documented in cancer cells.

EcDNA and BFB thus form the yin and yang of focal oncogene amplification. EcDNA results in high cell-to-cell variability of copy numbers, possibly with resistance to targeted treatment, high chromatin accessibility (Wu *et al.* 2019), enhancer hijacking (Helmsauer *et al.* 2020), lowered immune response (Wu *et al.* 2022), and worse outcomes for patients (Kim *et al.* 2020, 2024, Bailey *et al.* 2024). In contrast, BFB cycles result in a stable chromosomal amplification across different cells, increased immune response, and possibly improved outcomes (Raeisi Dehkordi *et al.* 2025). A proper exploration of the phenotypic differences between these two mechanisms requires good tools to distinguish the mechanisms in genomic data. Here, we present a tool for identifying BFB cycles reliably using long-read (Oxford Nanopore Technology) data.

BFBs are often marked by a ladder-like focal amplification and an excess of inverted duplications, or *foldback* inversions (see Materials and methods). Many researchers simply define BFB as genomic regions with an excess of these signatures (Bignell *et al.* 2007, Hadi *et al.* 2020, Keskus *et al.* 2026). However, because BFBs are precursors to other rearrangements including ecDNA formation, these signatures may not always distinguish true BFBs from ecDNA and other rearrangements (Raeisi Dehkordi *et al.* 2025). Other approaches have centered around mathematical exploration of BFB structures (Kinsella and Bafna 2012, Zakov *et al.* 2013, Li *et al.* 2023), and focus on a more stringent definition of BFB as one in which the bulk of the copy numbers and structural variants in a region can be explained by a BFB sequence. Zakov *et al.* (2013) provided an efficient algorithm for testing if a CN profile matches BFB exactly, and for generating BFB sequences given noisy copy number profiles (Zakov and Bafna 2015). Li *et al.* (2023) proposed a graph algorithm for generating BFB sequences in local genomic regions using integer linear programming (ILP). Raeisi Dehkordi *et al.* (2025) exploited these methods to generate candidate BFB sequences, and developed a method, OM2BFB to score candidates against the copy numbers and foldbacks observed using optical genome maps. The OM2BFB method showed excellent precision and recall compared to previous methods on cytogenetically validated examples.

This paper builds upon these ideas in two ways: (i) it develops mathematical properties and an ILP-based algorithm that automatically reconstructs the BFB sequence most consistent with both copy number and foldback data, and (ii) it works on Oxford Nanopore sequences, which allows it to identify important structural properties of BFB sequences. The resulting method, *BFBArchitect*, shows precision and recall that matches OM2BFB, but provides deeper insights into the nature of bridge formation, telomere reacquisition, and internal structural variation.

## 2 Materials and methods

### 2.1 Overview

**Figure 1:**
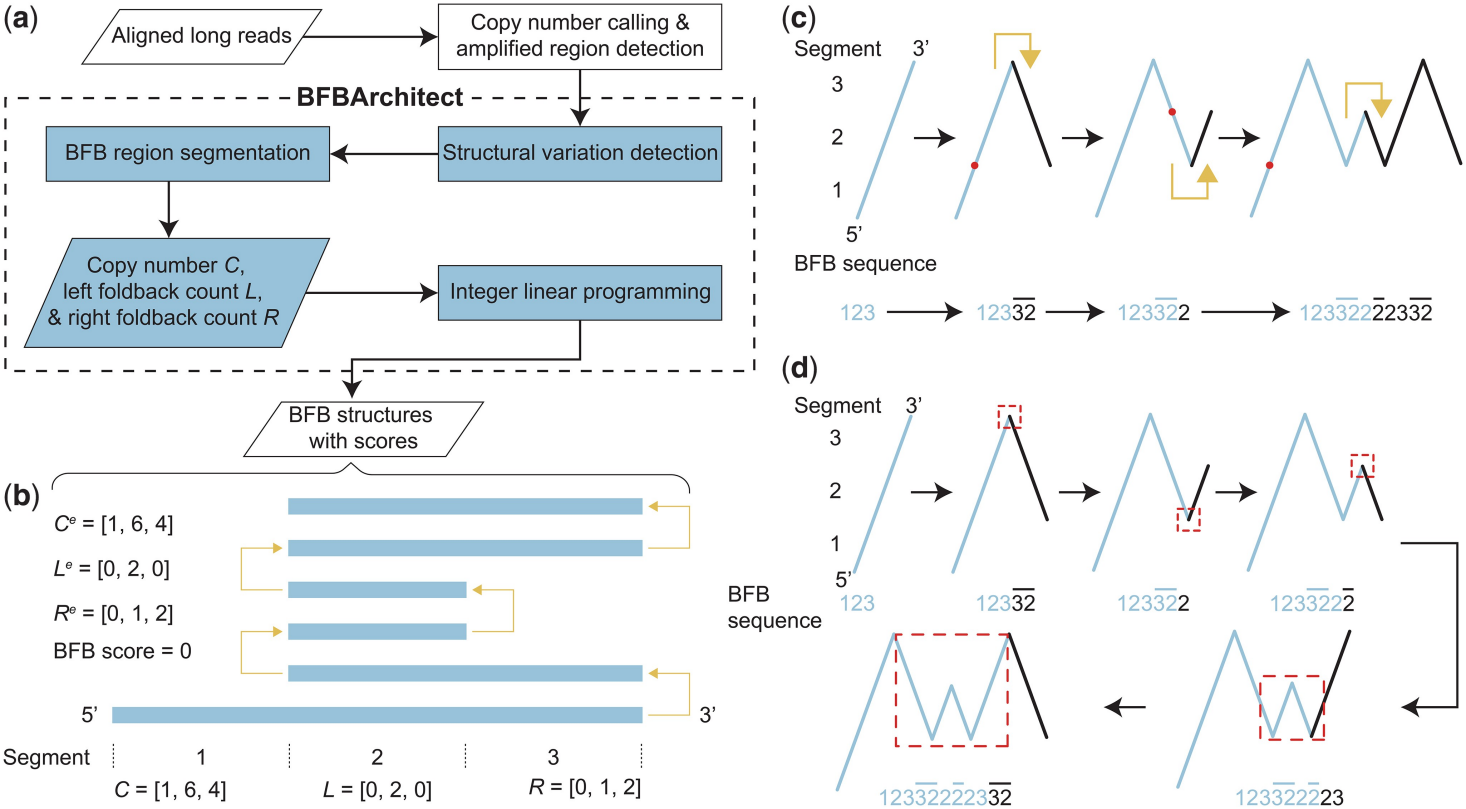
Overview of BFBArchitect. (a) Workflow of BFBArchitect. (b) Schematic representation of BFBArchitect output. (C,L,R) are observed vectors from the sequencing data, and $\left(C^{e},L^{e},R^{e}\right)$ are derived from the output BFB sequence, which consists of 1 copy of segment 1, 6 copies of segment 2, and 4 copies of segment 3. Also, the output BFB sequence contains 2 copies of left foldback $\bar{2}2$, 1 copy of right foldback $2\bar{2}$, and 2 copies of right foldback $3\bar{3}$, represented by arrows. (c) BFB cycles leading to the BFB sequence in (b). In each BFB cycle, the dot represents the start of the palindromic suffix derived by inverted duplication that generates the suffix following the foldback. The text below shows the exact BFB sequence generated in each BFB cycle. (d) Consecutive sequences that generate the BFB sequence in (b). Dashed boxes denote a (possibly empty) palindromic sequence. The text below shows the BFB sequence reconstructed at each timestep.

BFBArchitect takes aligned sequencing reads as input. After calling copy number variation and structural variants, and locating amplified regions using standard pipelines like CoRAL (Zhu *et al.* 2024), it automatically detects BFB candidates as genomic regions with high foldbacks and focal amplifications. Next, it partitions a candidate BFB region into *n* contiguous segments (Fig. 1a), based on CN shifts and foldback locations. BFBArchitect first uses foldback locations to partition the region. If a sharp change in CN has a distance >50 kb from all foldback locations, BFBArchitect uses the CN shift location for additional segmentation. BFBArchitect then extracts three vectors of size *n* from the sequencing data of the segmented region: a copy number vector C providing copy numbers of segments, a left foldback vector L providing left foldback counts on segments, and a right foldback vector R providing right foldback counts on segments. Note that (C,L,R) can be generated for a candidate region using standard pipelines, e.g. Severus (Keskus *et al.* 2026), CoRAL (Zhu *et al.* 2024), AmpliconSuite (Luebeck *et al.* 2024), and others, thus making BFBArchitect technology agnostic; however, in this work, we utilize ONT long reads, the lengths of which make it less likely to miss foldbacks.

Note that a BFB sequence also induces three vectors $\left(C^{e},L^{e},R^{e}\right)$. For example, $\alpha =\langle c\rangle 123\bar{32}2\bar{2}23\bar{32}$ consists of n=3 segments and induces: (i) $C^{e}=[1,6,4]$ that represents 1 copy of segment 1, 6 copies of segment 2, and 4 copies of segment 3; (ii) $L^{e}=[0,2,0]$ that represents 2 copies of left foldback connecting segment $\bar{2}$ to segment 2; (iii) $R^{e}=[0,1,2]$ that represents 1 copy of right foldback connecting segment 2 to segment $\bar{2}$ and 2 copies of right foldback connecting segment 3 to segment $\bar{3}$ (Fig. 1b).

To develop BFBArchitect, we first provide a novel combinatorial description of BFB sequences. Note that any BFB sequence can be constructed by iteratively appending subsequences in alternate directions. In Section 2.2, we describe a necessary and sufficient conditions to maintain the BFB sequence property while appending consecutive sequences. Thus in each step, there is a predictable change of copy number and foldbacks as a consecutive sequence is added. This allows us to develop an integer linear programming (ILP) optimization (see Section 2.3 and Section 2.4) that generates a BFB sequence with optimal concordance between the observed (C,L,R) and inferred $\left(C^{e},L^{e},R^{e}\right)$. A non-linear function (see Section 2.5) is finally applied to score the candidate BFB sequence. The optimized solution thus yields both a reconstructed BFB sequence and a score reflecting the discrepancy between the observed data and the reconstructed BFB sequence. The BFB sequence is output as a positive result if the score is below an empirical threshold; otherwise, the amplification is considered as non-BFB.

### 2.2 Modeling a BFB sequence

A BFB sequence is represented as an ordered concatenation of genomic segments, each labeled by its position along a chromosomal arm. Let the chromosome arm be composed of *n* ordered segments 1,2,…,n from 5′ end to 3′ end. Each segment i has a reverse complement $\bar{i}$, which is the segment obtained by duplicating and inverting segment i in a BFB cycle (Fig. 1c). The structural variant linking segment i to segment $\bar{i}$ is a *right foldback* denoted as $i\bar{i}$, and the structural variant linking segment $\bar{i}$ to segment i is a *left foldback* denoted as $\bar{i}i$.

Definition 1 (BFB template).

An n-segment* BFB template* is a chromosome arm without telomere, denoted as a string 12…n if BFB cycles occur on the q arm or $\bar{n(n-1)\ldots 1}$ if BFB cycles occur on the p arm.

Definition 2 (Consecutive sequence).

A* consecutive sequence* $c_{ij}$ is defined as a substring of the BFB template, i.e. i(i+1)…j for 1≤i≤j≤n, and its reverse complement is denoted by cij=j(j-1)…i. Note that a consecutive sequence does not contain foldbacks. For any j≤k≤n, ${\bar{c}}_{ik}$ is a* super consecutive sequence* of $c_{ij}$; for any 1≤k≤i, $c_{kj}$ is a super consecutive sequence of ${\bar{c}}_{ij}$.

Definition 3 (BFB sequence).

A string α is a* BFB sequence* if and only if it can be generated recursively as follows:

- **Base case:**$\alpha =c_{1n}=12\ldots n$ or α=c¯1n=n(n−1)…1.
- **Recursive case:**$\alpha =\beta x\bar{x}$, where βx is itself a BFB sequence, and β (that can be empty) and x x are concatenations of one or more consecutive sequences. Note that the last segment in β (if not empty) and the first segment in x are either two adjacent segments on a normal chromosome or reverse complements connected by a foldback inversion.

Lemma 1. *If* α=βx *is a BFB sequence, then* $\beta x\bar{x}$ *is a BFB sequence.*

**Proof.** By definition, a BFB sequence is generated by iterative suffix duplication and inversion. If α=βx is a valid BFB sequence, then duplicating the suffix x and appending its reverse complement $\bar{x}$ produce $\beta x\bar{x}$, which also satisfies the recursive definition of a BFB sequence.

Lemma 2. *Any prefix of a BFB sequence formed by removing a consecutive sequence suffix is a BFB sequence.*

**Proof.** Let $\alpha =\beta x\bar{x}$ denote a BFB sequence, and let $\alpha^{\prime }$ be an arbitrary prefix formed by removing a consecutive sequence suffix c, i.e. $\alpha =\alpha^{\prime }c$. Since c does not contain foldbacks and there is a foldback connecting x to $\bar{x}$, thus $\left|\bar{x}|\geq |c\right|$. When $\left|\bar{x}|>|c\right|$, let $\bar{x}=yc$, and $\alpha =\beta \bar{c}\bar{y}yc$ is a BFB sequence. Then, $\beta \bar{c}\bar{y}$ is a BFB sequence by definition, and $\alpha^{\prime }=\beta \bar{c}\bar{y}y$ is a BFB sequence according to Lemma 1. When $\left|\bar{x}|=|c\right|$, since c is a consecutive sequence that does not contain a foldback, we have $\bar{x}=c$. Then, $\alpha^{\prime }=\beta x$ is a BFB sequence by definition.

Theorem 1. *Let* α*denote a string and*c* denote a consecutive sequence.* αc*is a BFB sequence if and only if* α*is a BFB sequence and* ∃β,y*(possibly empty) such that* $\alpha =\beta \bar{c}\bar{y}y.$

**Proof.** (if) Let $\alpha =\beta \bar{c}\bar{y}y$ be a BFB sequence. Then, $\beta \bar{c}\bar{y}$ is a BFB sequence by definition, and $\alpha c=\beta \bar{c}\bar{y}yc$ is a BFB sequence by Lemma 1.

(only-if) Suppose αc is a BFB sequence. Denote $\alpha c=\beta x\bar{x}$. Since c is a consecutive sequence, it contains no foldback. Since there is a foldback between x and $\bar{x}$, we have |x|≥|c|. Thus, c must be a suffix of x, and we can write $\bar{x}=yc$ for some sequence y. Then, $\alpha c=\beta x\bar{x}=\beta \bar{c}\bar{y}yc$ is a BFB sequence. According to Lemma 2, $\alpha =\beta \bar{c}\bar{y}y$ is a BFB sequence.

Theorem 1 provides an algorithmic framework for constructing BFB sequences. In this work, we build a BFB sequence by iteratively appending consecutive sequences that satisfy Theorem 1 (Fig. 1d).

### 2.3 Reconstructing a BFB sequence using ILP

Building on the BFB model, we formulate an ILP approach to reconstruct a BFB sequence that best explains the observed copy numbers $C=[C_{1},C_{2},\ldots ,C_{n}]$, and the observed foldback counts, i.e. $L=[L_{1},L_{2},\ldots ,L_{n}]$ and $R=[R_{1},R_{2},\ldots ,R_{n}]$. Note that any BFB sequence induces increased copy numbers of certain segments and foldback SVs. The “best” BFB sequence minimizes the discrepancy between the observed copy numbers and foldback counts and the induced ones. Starting from the BFB template 12⋯n (or n⋯21), we build a BFB sequence by appending consecutive sequences in T timesteps, where $T=1+\sum_{i=1}^{n}L_{i}+\sum_{i=1}^{n}R_{i}$. In each timestep, we add exactly one consecutive sequence.

Let $c_{ij}^{\left(d\right)}[t]\in \{0,1\}$ denote a binary variable that equals 1 if the consecutive sequence $c_{ij}^{\left(d\right)}$ is appended at timestep t, and 0 otherwise, where 1≤i≤j≤n, d∈{+,−}, and t=1,2,…,T. Note that $c_{ij}^{\left(+\right)}$ and $c_{ij}^{\left(-\right)}$ represent consecutive sequences $c_{ij}$ and ${\bar{c}}_{ij}$ described in Definition 2. Then, we can derive a BFB sequence that consists of T consecutive sequences, which induce the estimated copy numbers $C^{e}=[C_{1}^{e},C_{2}^{e},\ldots ,C_{n}^{e}]$ and estimated foldback counts $L^{e}=[L_{1}^{e},L_{2}^{e},\ldots ,L_{n}^{e}]$ and $R^{e}=[R_{1}^{e},R_{2}^{e},\ldots ,R_{n}^{e}]$. For each segment k∈{1,2,…,n}, we have $C_{k}^{e}=\sum_{i=1}^{k}\sum_{j=k}^{n}\sum_{t=1}^{T}\left(c_{ij}^{\left(+\right)}[t]+c_{ij}^{\left(-\right)}[t]\right)$, $L_{k}^{e}=\sum_{j=k}^{n}\sum_{t=2}^{T}c_{kj}^{\left(+\right)}[t]$, and $R_{k}^{e}=\sum_{i=1}^{k}\sum_{t=2}^{T}c_{ik}^{\left(-\right)}[t]$. To minimize the discrepancies between (C,L,R) and $\left(C^{e},L^{e},R^{e}\right)$, we introduce non-negative error variables $\varepsilon_{k}^{c},\varepsilon_{k}^{l},\varepsilon_{k}^{r}$ representing the absolute deviations between the observed and estimated values for segment k. Thus, our ILP objective function is as follows:


$$\begin{array}{cc} \text{minimize} & \sum_{k=1}^{n}\left(\varepsilon_{k}^{c}+\varepsilon_{k}^{l}+\varepsilon_{k}^{r}\right)\\ \text{subject}\;\text{to} & \varepsilon_{k}^{c}\geq |C_{k}-C_{k}^{e}|,\;\varepsilon_{k}^{l}\geq |L_{k}-L_{k}^{e}|,\;\varepsilon_{k}^{r}\geq |R_{k}-R_{k}^{e}|,\\ & \varepsilon_{k}^{c},\varepsilon_{k}^{l},\varepsilon_{k}^{r}\geq 0\;\forall k=1,2,\ldots ,n. \end{array}$$

### 2.4 ILP constraints

To ensure that the ILP framework follows our BFB model, which leads to a canonical BFB structure, the following constraints must be satisfied, with details in Supplementary Methods 1, available as supplementary data at *Bioinformatics* online:

1. At the first timestep, if the BFB region is on the q arm, $c_{1n}^{\left(+\right)}[1]=1$; otherwise, $c_{1n}^{\left(-\right)}[1]=1$.
2. At each timestep t, exactly one consecutive sequence is added, i.e.
  $$\sum_{i=1}^{n}\sum_{j=i}^{n}\left(c_{ij}^{\left(+\right)}[t]+c_{ij}^{\left(-\right)}[t]\right)=1\;\forall t=1,2,\ldots ,T.$$
3. The directions of adjacent consecutive sequences alternate, i.e.
  $$\sum_{i=1}^{n}\sum_{j=i}^{n}\left(c_{ij}^{\left(+\right)}[t]+c_{ij}^{\left(+\right)}[t+1]\right)-\sum_{i=1}^{n}\sum_{j=i}^{n}\left(c_{ij}^{\left(-\right)}[t]+c_{ij}^{\left(-\right)}[t+1]\right)=0\;\;\;\;\;\;\;\;\;\;\;\;\;\;\;\;\;\;\;\;\;\;\;\;\;\;\;\;\;\;\;\;\;\;\;\;\;\;\;\;\;\;\;\;\;\;\;\;\;\forall t=1,\ldots ,T-1.$$
4. When adding $c_{ij}^{\left(d\right)}$ at timestep t, i.e. $c_{ij}^{\left(d\right)}[t]=1$, two conditions must be satisfied to guarantee the resulting string is a BFB sequence: (1) A super consecutive sequence is added at a previous timestep $t^{\prime }$; (2) The consecutive sequences added between timesteps $t^{\prime }$ and t (excluding both endpoints) form a palindrome. Note that an empty string is also a palindrome.

### 2.5 Scoring the candidate BFB sequence

We devised a non-linear BFB score function to measure the consistency between the reconstructed BFB sequence and the observed information in the amplified region. BFBArchitect partitions an amplified region into n contiguous segments. Segment i has a coverage $S_{i}$, and normal regions without amplification have an average coverage S. We can calculate the observed copy number $C_{i}=\text{round}(2\times \frac{S_{i}}{S}-1)$ for segment i. Similarly, we can calculate the observed left foldback count $L_{i}=\text{round}(2\times \frac{S_{i}^{l}}{S}-1)$ and the observed right foldback count $R_{i}=\text{round}(2\times \frac{S_{i}^{r}}{S}-1)$, where $S_{i}^{l}$ and $S_{i}^{r}$ are the numbers of supporting reads for left and right foldbacks on segment i. We then have the observed count vectors $C=[C_{1},C_{2},\ldots ,C_{n}]$, $L=[L_{1},L_{2},\ldots ,L_{n}]$, and $R=[R_{1},R_{2},\ldots ,R_{n}]$. BFBArchitect takes (C,L,R) as input for the ILP optimization and reconstructs a BFB sequence, which induces the estimated count vectors $C^{e}=[C_{1}^{e},C_{2}^{e},\ldots ,C_{n}^{e}]$, $L^{e}=[L_{1}^{e},L_{2}^{e},\ldots ,L_{n}^{e}]$, and $R^{e}=[R_{1}^{e},R_{2}^{e},\ldots ,R_{n}^{e}]$. To measure the BFB sequence, we devised a score function as follows:


$$\begin{array}{cc} & \text{BFB}\;\text{score}=\sum_{i=1}^{n}\frac{\left|C_{i}-C_{i}^{e}\right|}{C_{i}^{e}}+\frac{\text{Dis}(L,L^{e})+\text{Dis}(R,R^{e})}{n}\\ & +\frac{\sum_{i=1}^{n}I(L_{i},L_{i}^{e})+I(R_{i},R_{i}^{e})}{2}+\sum_{i=1}^{n}\frac{\left|(2\times \frac{S_{i}}{S}-1)-C_{i}\right|}{C_{i}},\\ & \text{where}\;\text{Dis}(X,Y)=∥X-Y{∥}_{2}\;\text{and}\;I(x,y)=\{\begin{array}{cc} 1 & \text{if}\;x=0,y>0\\ 0 & \text{otherwise} \end{array}. \end{array}$$

The BFB score is the sum of four terms describing the discrepancies between the input and output. The first two terms represent the discrepancies between (C,L,R) and $\left(C^{e},L^{e},R^{e}\right)$. The third term penalizes the foldbacks in the output BFB sequence, which are not supported by the observed foldback counts. The last term represents the error for estimating copy numbers from the sequencing data. Overall, a lower BFB score indicates a stronger agreement between observed data and the reconstructed BFB sequence, suggesting that the region is highly likely to be a true BFB amplicon; whereas a higher score indicates deviation from canonical BFB structures. Perfect (C,L,R) patterns can generate a BFB sequence with a BFB score of 0 (Fig. 1b).

### 2.6 Handling deletions in BFB

When a deletion occurs in the BFB sequence, the observed copy numbers C are underestimated if the deletion is ignored in the BFB reconstruction. We handle deletions by refining C to $C^{r}$, reconstructing a BFB sequence with input $\left(C^{r},L,R\right)$, and then correcting the BFB sequence by incorporating the deletion. Assume that a segment i has a coverage $S_{i}$ and length $\delta_{i}$. Denote the average coverage of normal regions without amplification as S. If there is a deletion with length $\delta_{d}$ in segment i, the coverage $S_{i}=\frac{l_{i}}{\delta_{i}}$ is underestimated in the first place, when we divide the total length $l_{i}$ of reads covering segment i by the segment length $\delta_{i}$. To correct the coverage, we should use a shorter segment length due to deletion, i.e. $\delta_{i}-\delta_{d}$. Hence, the corrected coverage for segment i is $\frac{\delta_{i}}{\delta_{i}-\delta_{d}}\times S_{i}$. We refine the copy number by $C_{i}^{r}=\text{round}(2\times \frac{\delta_{i}}{\delta_{i}-\delta_{d}}\times \frac{S_{i}}{S}-1)$. In this way, we can correct the copy numbers that are underestimated due to deletions, and the re-estimated copy numbers $C^{r}$ provide more accurate information for BFB reconstruction.

## 3 Results

### 3.1 Datasets

We simulated BFB sequences by modeling a telomeric break followed by a sequence of prefix inverted duplications (see details in Supplementary Methods 2, available as supplementary data at *Bioinformatics* online). Deletions with an average size of 200 kb were introduced to add complexity in the BFB sequences. Next, we used NanoSim (Yang *et al.* 2017) to sample ONT reads from the BFB sequences using average coverage 15× with an error rate of 3.79% and a mean length of 5267 bp. These were mixed with ONT reads from whole-genome sequencing samples with average coverage 15× to create samples with BFB amplification labeled as BFB(+). A total of 62 BFB(+) samples were generated. Additionally, 134 control samples labeled as BFB(−) were generated using ecSimulator (Zhu *et al.* 2024) with default parameters (Supplementary Methods 2, available as supplementary data at *Bioinformatics* online), which simulates non-BFB focal amplifications using a variety of mechanisms, including ecDNA and tandem duplication. Some of the ecDNA generation methods, such as 2-foldback (Passananti *et al.* 1987), also include some foldback reads in addition to high copy number amplification. On average, the simulated BFB(+) and BFB(−) samples contained 4.52 and 2.82 foldbacks, respectively, and they both had one additional SV other than foldbacks (Fig. 1, available as supplementary data at *Bioinformatics* online).

We also have nine BFB(+) and nine BFB(−) cases from cell lines that were validated previously. The validation was based on one of two criteria: (i) An amplification was a BFB if and only if metaphase FISH for the amplified probe displayed an HSR (homogeneously staining region) only *on the native chromosome*. Otherwise, it was deemed to be BFB(−). (ii) When FISH was not available, an OM2BFB designation as BFB(+) was considered to be an orthogonal validation because of its previously shown accurate performance (Raeisi Dehkordi *et al.* 2025). Overall, eight amplifications were cytogenetically validated by FISH, and 10 amplifications were technically validated by OM2BFB (the detailed results are provided in Table 1, available as supplementary data at *Bioinformatics* online).

### 3.2 BFBArchitect accurately identifies BFB amplifications

**Figure 2:**
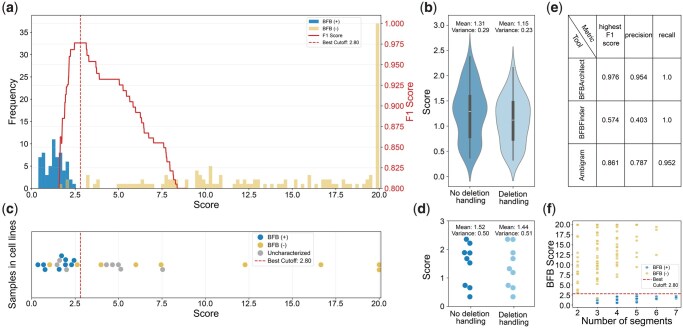
Performance of BFBArchitect on simulations and cell lines. (a) BFB score distribution of 62 simulated BFB(+) and 134 BFB(−) samples. The best score cutoff 2.80 achieves the highest F1 score 0.976. (b) BFB(+) scores with and without deletion handling in simulations. (c) BFB score distribution of nine BFB(+), nine BFB(−), and eight uncharacterized cases from eleven cancer cell lines. (d) BFB(+) scores with and without deletion handling in cell lines. (e) The best F1 score and the corresponding precision and recall achieved by running BFBArchitect, BFBFinder, and Ambigram on all simulated samples. (f) The distribution of BFBArchitect-generated BFB scores over the number of amplified segments.

On the 196 simulated samples, a BFB score cutoff of 2.80 effectively separated positive and negative examples with 98.47% accuracy (F1 score = 0.976, Fig. 2a). The mean scores of 1.31 and 13.64 were well separated, and only 2% of the negative samples had a score <3. Notably, the fraction of foldback SVs was not very different between positive and negative examples. The SV correction for deletions also improved the BFB(+) scores from a median of 1.34 (IQR = (0.82, 1.64)) to 1.16 (IQR = (0.76, 1.52)) (Fig. 2b).

Consistent with the simulations, the cutoff of 2.80 also separated the validated BFB(+) from BFB(−) samples (Fig. 2c). The results matched BFB predictions on the same cell lines made with optical genome mapping data (Raeisi Dehkordi *et al.* 2025), and almost perfectly separated the positive and negative examples, with the discrepancies explained below. As with simulations, the SV correction for deletion further improved the BFB(+) scores from a median of 1.73 to 1.37 (Fig. 2d). Given aligned reads and amplified regions, BFBArchitect efficiently reconstructed BFB sequences in all cases from simulations and cell lines within 1300 s (Fig. 2, available as supplementary data at *Bioinformatics* online). Note that the BFB(+) cases from simulations and cell lines have an average segment CN of 7.08 and 14.06, resulting in BFB sequences of average lengths 20.90 and 38.78 (in the number of segments), respectively.

We compared BFBArchitect to the other two tools, BFBFinder (Zakov *et al.* 2013) and Ambigram (Li *et al.* 2023), for reconstructing BFB sequences. BFBFinder takes only the observed copy numbers as input and outputs a BFB sequence if the input is consistent with the BFB model within a specified error weight. Ambigram is a graph-based method that takes copy numbers and structural variants as input, generates candidate substrings using a complex ILP model, and then assembles these substrings into a BFB sequence with an ILP score that measures the discrepancies between the input data and the output BFB sequence. For a fair comparison, we input identical copy numbers and structural variants from simulated data, including all BFB(+) and BFB(−) samples, into BFBArchitect, BFBFinder, and Ambigram. We ran BFBFinder (parameters: -v -a -e=PoissonErrorModel) with the weight (parameter: -w) ranging from 0.5 to 1.0 with a step size of 0.05 and calculated the F1 score under each weight. We ran Ambigram with the default parameters and calculated the F1 scores with thresholds spanning all possible ILP scores output by Ambigram. Finally, we ran BFBArchitect with the customized scoring and computed the F1 score for each threshold ranging from 0 to 10 with a step size of 0.01. BFBArchitect achieved the best F1 score 0.976, which was much higher than the F1 score 0.574 of BFBFinder (precision = 0.403, recall = 1.0, weight = 0.85) and the F1 score 0.861 of Ambigram (precision = 0.787, recall = 0.952, ILP cutoff = 35.5) (Fig. 2e and Tables 2–4, available as supplementary data at *Bioinformatics* online).

Similarly, on the cell lines, BFBArchitect (precision = 0.818, recall = 1.0) outperformed BFBFinder (precision = 0.556, recall = 0.625) and Ambigram (precision = 0.667, recall = 0.75) on these data (Tables 5–7, available as supplementary data at *Bioinformatics* online). The BFB signatures consist of characteristic (ladder-like) CN patterns as well as foldbacks. BFBFinder only utilizes CN patterns to make its predictions, not foldback reads. The two-step mechanism in Ambigram sometimes discards a few substrings that cannot be integrated into a BFB sequence, leading to a relaxed reconstruction of BFB. The CN-only input in BFBFinder and the relaxation in Ambigram resulted in underperformance of BFB reconstruction compared to BFBArchitect.

While BFBArchitect works well, the actual BFB score has a small positive correlation with the variable number of segments in BFB sequences in both BFB(+) and BFB(−) samples (Fig. 2f). This is a common feature of discrepancy-based functions. Notably, all real data that we have analyzed never had more than 10 segments, and the scores were well separated in that range. As more data is acquired, we will consider normalizing the score function for a higher number of segments.

**Figure 3:**
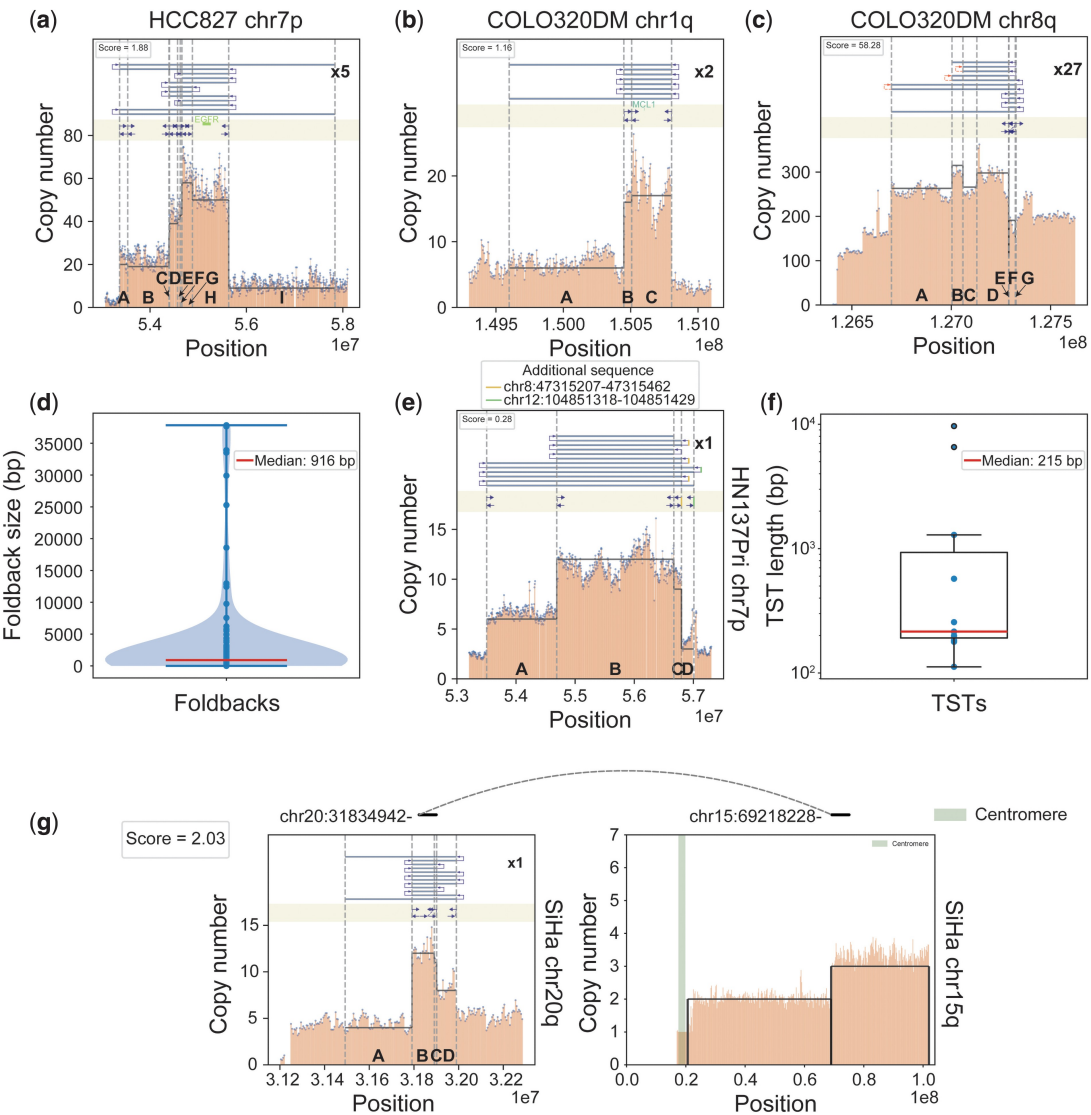
BFB characteristics identified by BFBArchitect. (a) A true positive BFB case from the HCC827 cell line. Note that segments 1,2,3… are denoted by A,B,C… in this and all the following plots of BFB sequences. (b) A false positive case from the COLO320DM cell line. (c) A true negative case from the COLO320HSR cell line. (d) Distribution of sizes of foldback inversions. (e) A BFB case with additional sequences between foldback junctions from the HN137Pri cell line. (f) Distribution of the lengths of additional sequences (TSTs: Tandem Short Templates) between foldback junctions. (g) Recapture of telomere in a BFB case from the SiHa cell line.

BFBArchitect can generate a BFB sequence down to the base-pair level that explains the copy numbers and foldbacks. We applied it to previously confirmed BFB amplicons. BFBArchitect reconstructed the *EGFR* BFB amplification in the lung cancer cell line HCC827 (Fig. 3a). Similar structures were predicted on eight other BFB(+) cases, along with a correct identification of BFB. The only discrepant calls were two “false-positive” identifications for matched chr1 amplifications in the cell lines COLO320DM and COLO320HSR (Fig. 3b and Fig. 3, available as supplementary data at *Bioinformatics* online). Both showed an HSR at the native location, but remarkably, also at a non-native location (Raeisi Dehkordi *et al.* 2025), thereby breaking our strict criterion and labeled as BFB(−) cases. However, these two examples are widely investigated as BFB by experimentalists (e.g. Shimizu *et al.* 2005), and are at least of BFB origin. Except for these two examples, BFBArchitect showed perfect precision and recall on the cell lines. We also note that extensive foldbacks are not a reliable indicator of BFB. The chr8q amplification in COLO320DM (Fig. 3c) is foldback-enriched but known to be ecDNA. Despite the extensive foldbacks, BFBArchitect could not reconstruct a concordant BFB sequence and correctly gave a worse score.

### 3.3 BFBArchitect reconstructs BFB sequences characterized by imperfect foldbacks

The BFBArchitect reconstruction provides a plausible sequence of BFB and provides an opportunity to better study its structural features. Expectedly, the sequence is dominated by foldbacks. However, for the most part, the foldbacks are not perfect hairpins (Li *et al.* 2023, Rodriguez *et al.* 2024, Raeisi Dehkordi *et al.* 2025). Rodriguez *et al.* (2024) describe three types of foldbacks, where type I represents an imperfect right foldback (respectively, left foldback) to be of the form $AB\bar{A}$ (respectively, $\bar{A}\bar{B}A,$ representing fusion with sister chromatids). We observed large variation in the length |B| of the overhang (median 916) (Fig. 3d). These data are consistent with a model where bridge formation is preceded by unbalanced nuclease digestion of the free ends (Rodriguez *et al.* 2024). The large overhang lengths could also indicate anaphase bridge formation prior to complete replication of the sister chromatid.

In a small number of cases, particularly in the Head and Neck cancer line HN137 (from both primary and metastatic resections), we observed additional sequences between the foldback junctions (e.g. Fig. 3e). These regions varying in length from $∼10^{2}$ to $10^{4}$ bp (Fig. 3f and Fig. 4, available as supplementary data at *Bioinformatics* online), all mapped to distant non-repetitive genomic loci, corresponding to type II and III events described by Rodriguez *et al.* (2024) and reminiscent of template switching DNA replication errors due to MMBIR (Umbreit *et al.* 2020). Despite heterogeneity between breakpoints, all reads supporting a single foldback junction carried an identical breakpoint sequence, consistent with a model that each break occurred only once during BFB cycle. The BFB sequences also contain additional SVs not limited to foldbacks. We observed a deletion of the *SHANK2* gene in the BFB region of HN137 (Pri- and Met-), a translocation in COLO829, and a duplication in HCC827 (Figs 5–7, available as supplementary data at *Bioinformatics* online).

### 3.4 BFBArchitect provides insight into BFB-cycle termination

An initial bridge formation and breakage is a precursor to additional genome instability, including chromothripsis and ecDNA formation (Ly *et al.* 2019, Umbreit *et al.* 2020). However, BFB cycles also result in stable chromosomal amplifications described here, by reacquiring telomeres to create a stable chromosome. How multiple BFB cycles reacquire the telomere is a longstanding problem since 1980s (McClintock 1983) that is incompletely understood. Previous results have suggested resolution of a telomere crisis through a translocation event (Meltzer *et al.* 1993) or through neo-telomere formation (Flint *et al.* 1994). However, these mechanisms have not been revealed in the context of BFB cycles. In the BFB(+) examples that we looked at, we observed translocation of the BFB sequence end to a distal region, but did not observe any telomeric repeats in BFB sequence reads.

For example, in the chr20q BFB sequence in the cell line SiHa, a translocation event precisely led to chr15q, with a partial aneuploidy of chr15q (Fig. 3g). This is consistent with an unbalanced translocation joining the BFB-sequence to chr15q telomere. Other translocations were more complex, including unbalanced translocations that did not lead directly to the telomere (Fig. 8, available as supplementary data at *Bioinformatics* online), and a translocation bridge amplification (Lee *et al.* 2023) (Fig. 9, available as supplementary data at *Bioinformatics* online). Thus, a stable BFB amplification could be a precursor to a more complex chromosomal amplification.

## 4 Discussion

BFBArchitect works with an abstract and simple input format defined by copy numbers and left and right foldbacks. Such data is readily available from many structural variant and copy number calling tools. Therefore, BFBArchitect should be agnostic to the sequencing methodology used. However, short reads are often not able to call foldbacks because of the complex heterogeneity at breakpoints shown in this paper and previous work (Rodriguez *et al.* 2024, Raeisi Dehkordi *et al.* 2025). Previous results have shown that short-read-based BFB detection is precise but has lower sensitivity (Raeisi Dehkordi *et al.* 2025). Therefore, we work with ONT in this paper. Extension to PacBio and other technologies should be straightforward, and will be developed in ongoing work.

The main technical contribution of BFBArchitect is to directly identify a BFB sequence that “best” explains the observed copy numbers and foldbacks using a novel characterization and ILP formation. Multiple sequences may be consistent with the data, and in future work, we plan to extend the methods to generate multiple BFB sequence candidates. We note that our reconstructed sequences are consensus representations of BFBs derived from bulk cell data. In general, BFBs are stable chromosomal amplifications with low cell-to-cell heterogeneity (Raeisi Dehkordi *et al.* 2025). However, some heterogeneity may occur due to additional SV in a subset of cells. These will show up as unexplained copy numbers in the reconstruction. In future work, we will modify our software to include a list of SVs that are not explained by the current reconstruction, and their copy numbers. Tumor purity is also an issue, but its impact on the BFB structure will mostly be to lower the average copy number, and we have not observed any direct impact. As our knowledge of true BFB(+) samples grows, we will also use BFBArchitect to better understand the repair pathways utilized in BFB formation, and the mechanisms of telomere acquisition.

The initial bridge formation and breakage during anaphase create instability. In some cases, it can drive ecDNA formation and chromothripsis. In other cases, it leads to a sequence of BFB cycles followed by telomere reacquisition, resulting in a stable chromosomal amplification, which we define as a BFB. However, sometimes the signatures of multiple amplification mechanisms can coexist. Other methods (Luebeck *et al.* 2024, Zhu *et al.* 2024) take the amplified region and build an amplicon graph, from which we can extract cycles corresponding to ecDNA, or pass it to BFBArchitect to reconstruct a BFB. In ongoing work, we will test this decomposition, which allows us to predict overlapping BFB and ecDNA events, and we will extend it to other amplification mechanisms. We anticipate that distinguishing between ecDNA and chromosomal amplifications mediated by BFB will be useful in distinguishing cancer samples for prognostic purposes. BFBArchitect makes a contribution to this by providing definitive identification of one type of chromosomal amplification (HSR). HSRs can also be formed by ecDNA re-integrating into the chromosome (Nathanson *et al.* 2014); however, those HSRs are considered to be less stable (Song *et al.* 2022), and we speculate that they will be phenotypically similar to ecDNA. Thus, identification of BFB-mediated focal amplifications provides prognostic information for targeted therapy success.

## Supplementary Material

btag225_Supplementary_Data

## Data Availability

The BFBArchitect source code is publicly available under an open-source license at https://github.com/AmpliconSuite/BFBArchitect. The repository includes detailed documentation, example datasets, and scripts for simulating BFB(+) samples and reproducing key results presented in this study. The simulated BFB(−) samples were generated by running the code at https://github.com/AmpliconSuite/ecSimulator using modules for focal amplification simulation, and nanopore read simulation (Supplementary Methods 2, available as supplementary data at *Bioinformatics* online).
