# Supplementary material for: Detecting and reconstructing breakage-fusion-bridge cycles from long-read sequencing using BFBArchitect
Source: Bioinformatics. 2026 Jul 7;42(Suppl 1):btag225. doi: 10.1093/bioinformatics/btag225 (PMC13340218; doi:10.1093/bioinformatics/btag225)
Supplement: btag225_Supplementary_Data [file btag225_supplementary_data.zip › Bafna.170.sup.1.pdf]

Supplementary Information for: Detecting and reconstructing breakage-fusion-bridge cycles from long-read sequencing using BFBArchitect

Supplementary Figures

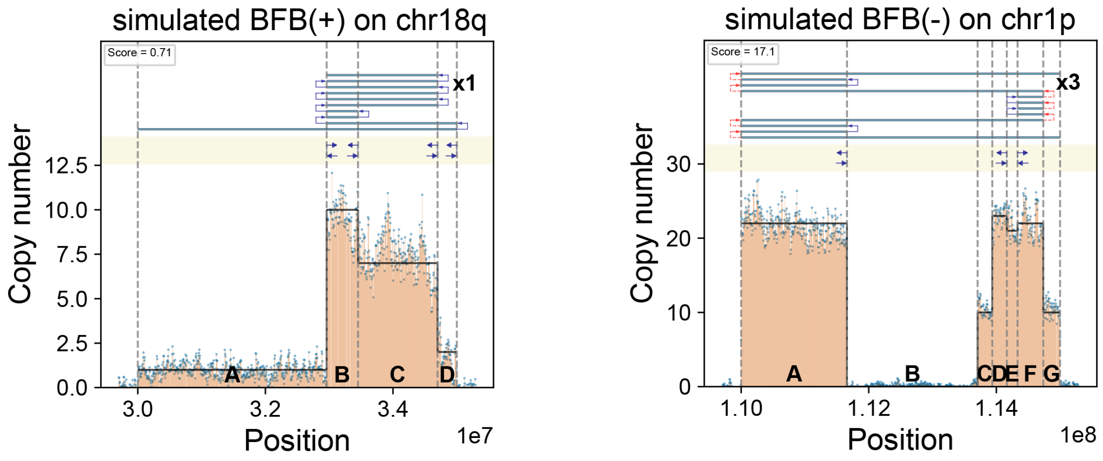

Supplementary Figure S1: **Examples of BFB(+) and BFB(-) simulations.**

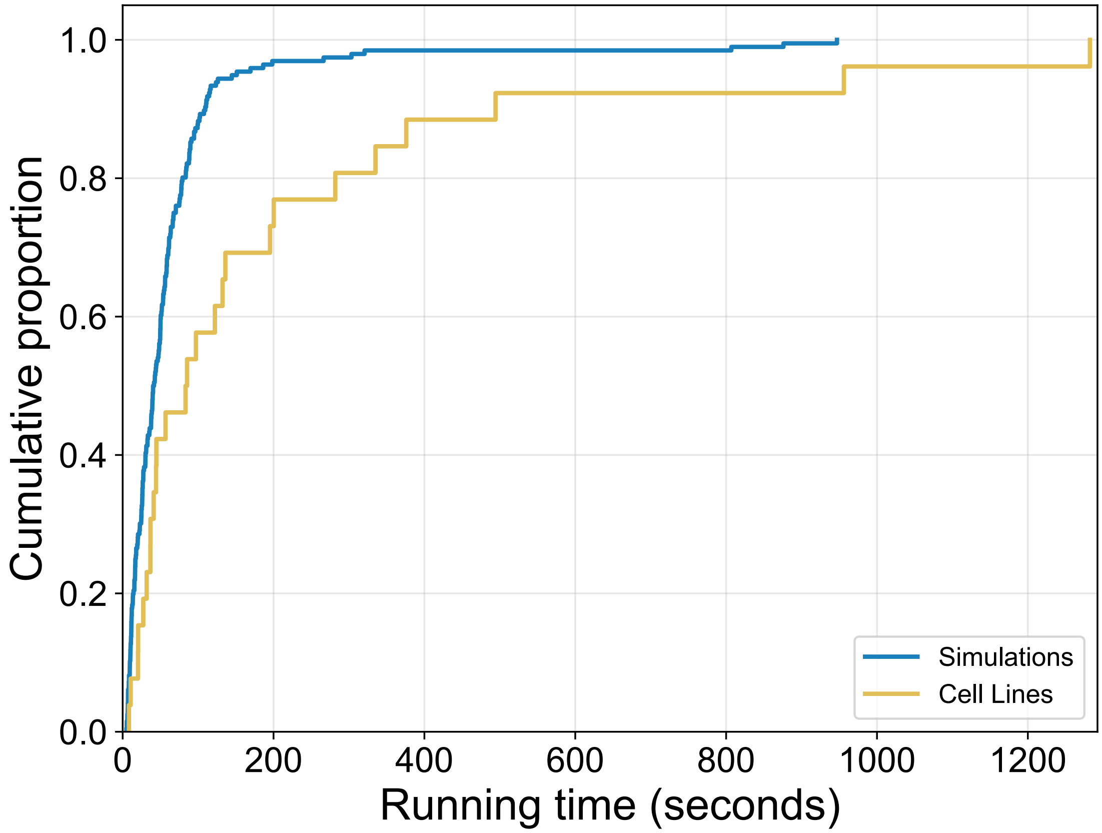

Supplementary Figure S2: **Cumulative running time of all simulated and cell line cases tested on a Linux machine (Intel Xeon X5680 @ 3.33 GHz, 128 GB RAM) running Ubuntu 16.04.6 LTS.**

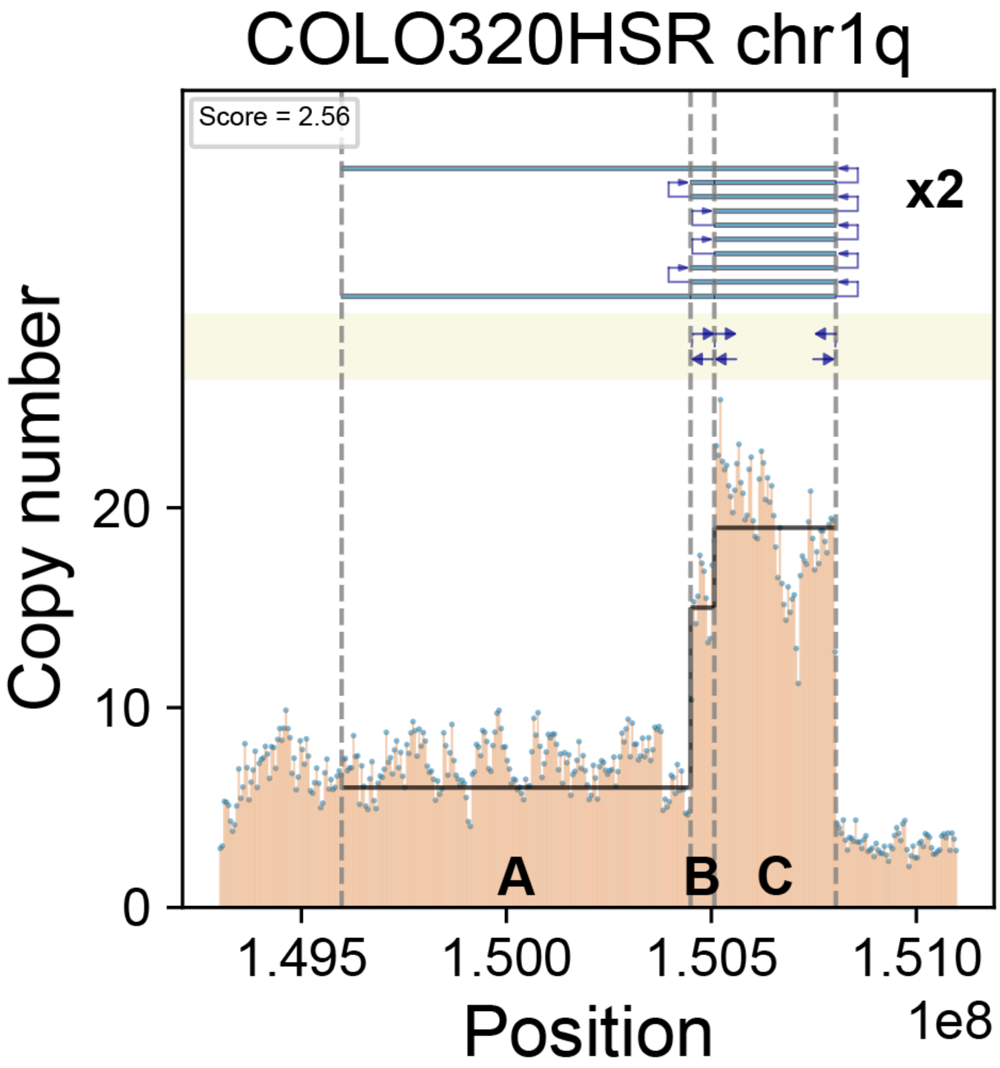

Supplementary Figure S3: **A false positive case from the COLO320HSR cell line.** The amplicon is in the isogenic region of the false positive from COLO320DM in Fig. 3b.

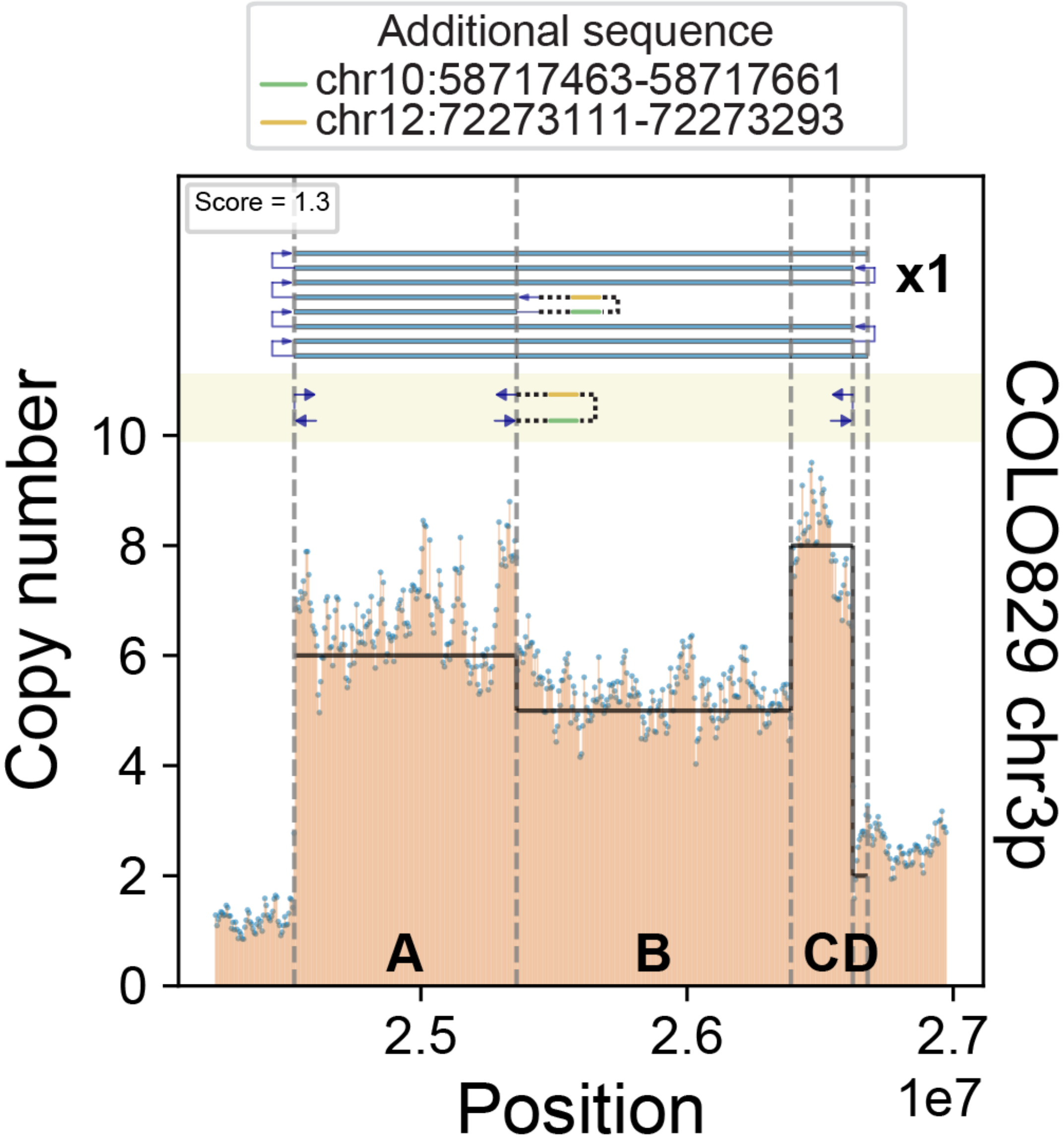

Supplementary Figure S4: **Additional sequences in a BFB amplicon from COLO829.** There are two additional sequences from chr10 and chr12 between foldback junctions on chr13 in the COLO829 cell line.

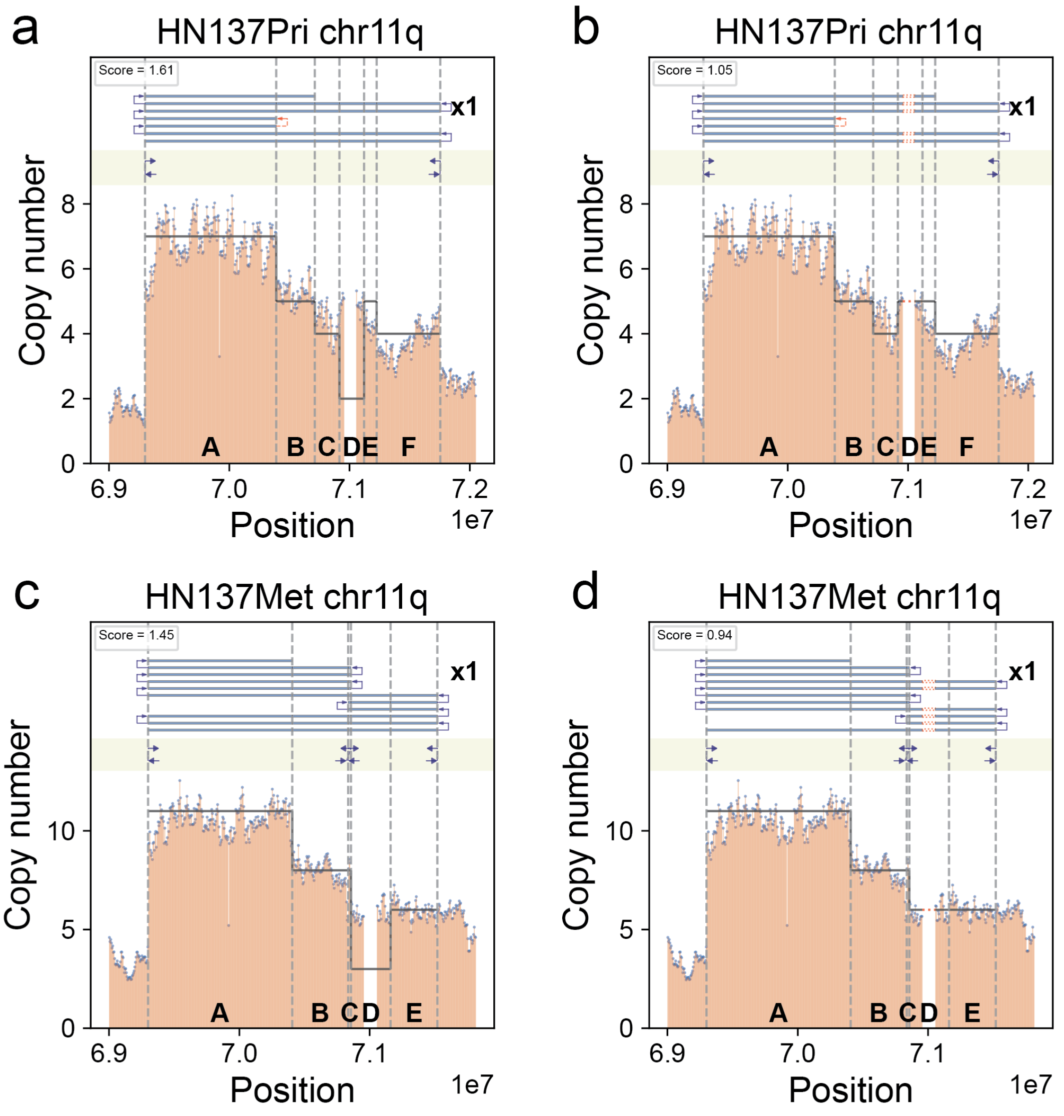

Supplementary Figure S5: **Deletion in isogenic BFB cases from HN137Pri (a) and HN137Met (c).** The deleted region chr11:70955695-71055697 includes *SHANK2* gene, which produces SHANK2 protein for proper brain function. Deletion handling improves the BFB score in both HN137Pri **(b)** and HN137Met **(d)**.

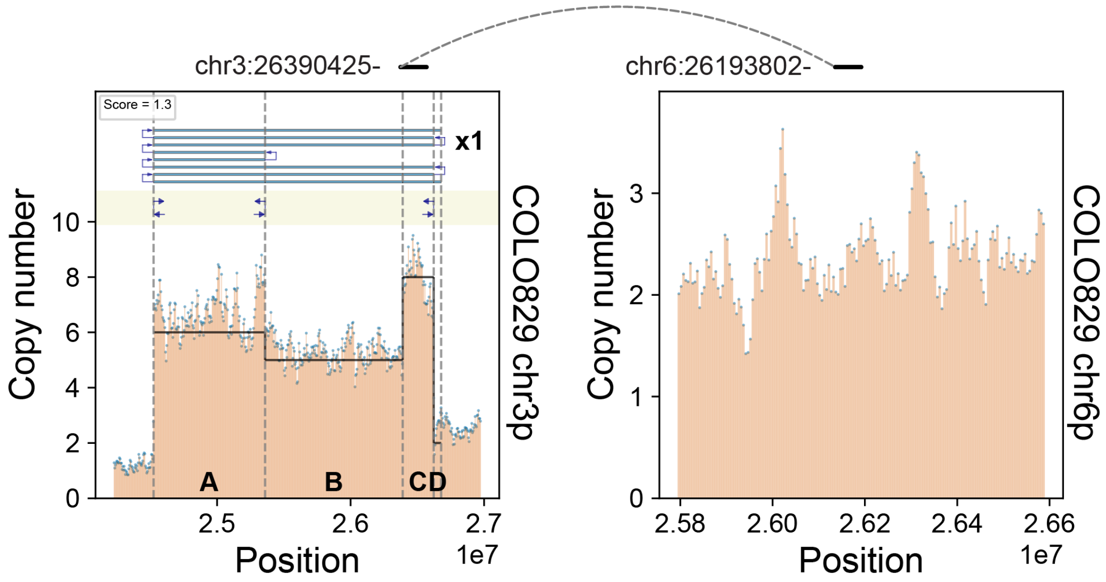

Supplementary Figure S6: **Translocation in a BFB amplicon from COLO829.** A translocations link the BFB region on chr3 to the p-arm on chr6 towards its centromere.

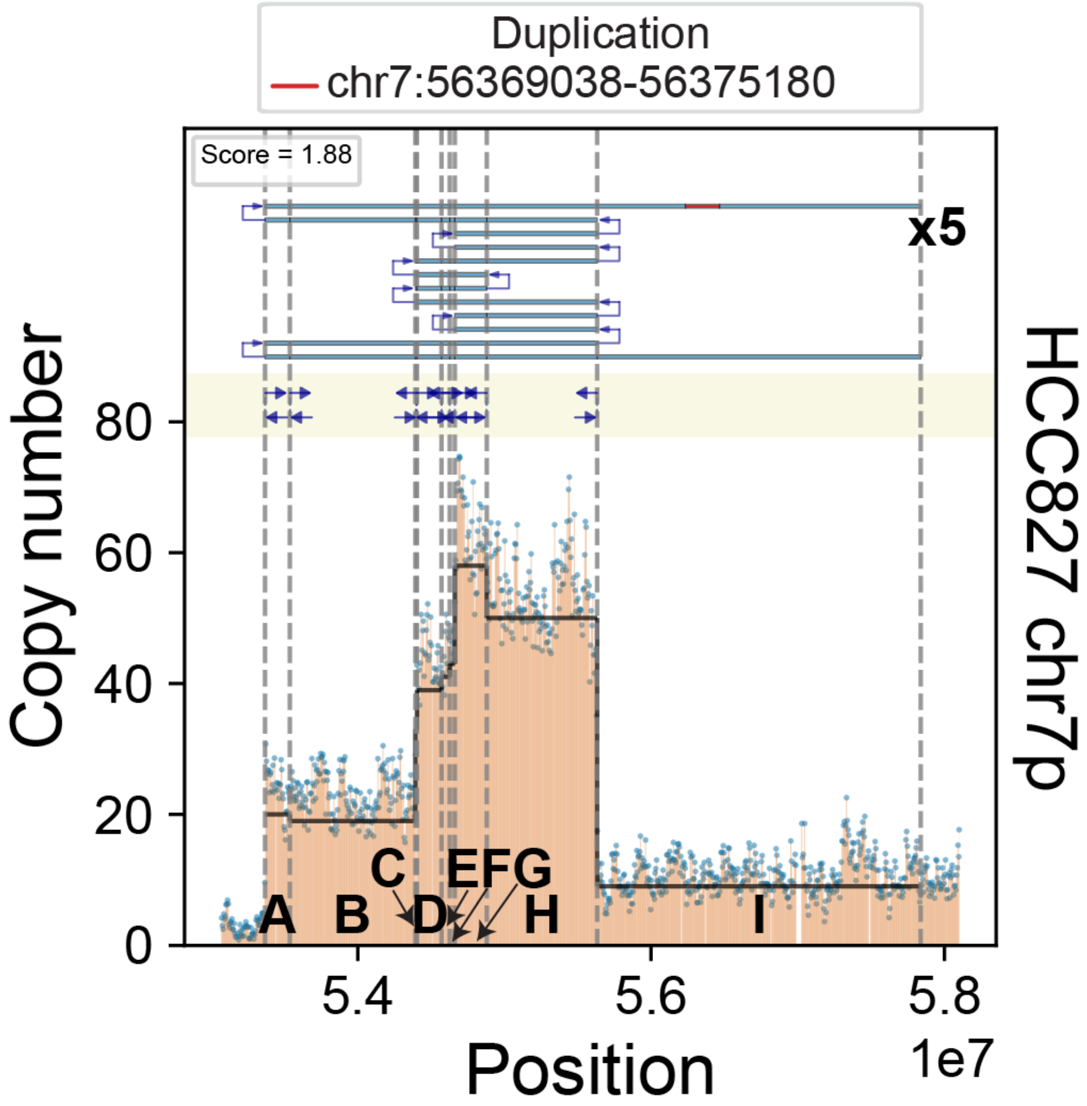

Supplementary Figure S7: Duplication in a BFB case from the HCC827 cell line.

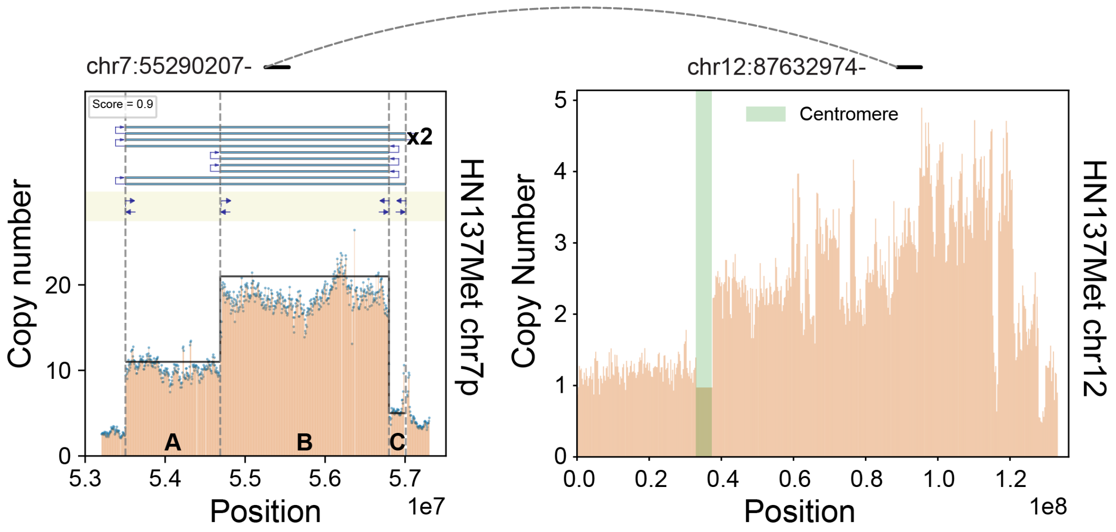

Supplementary Figure S8: Translocation from a BFB region on chr7 to chr12 in HN137Met. The translocation points to the q-arm of chr12, but it does not directly lead to the telomeric region on chr12.

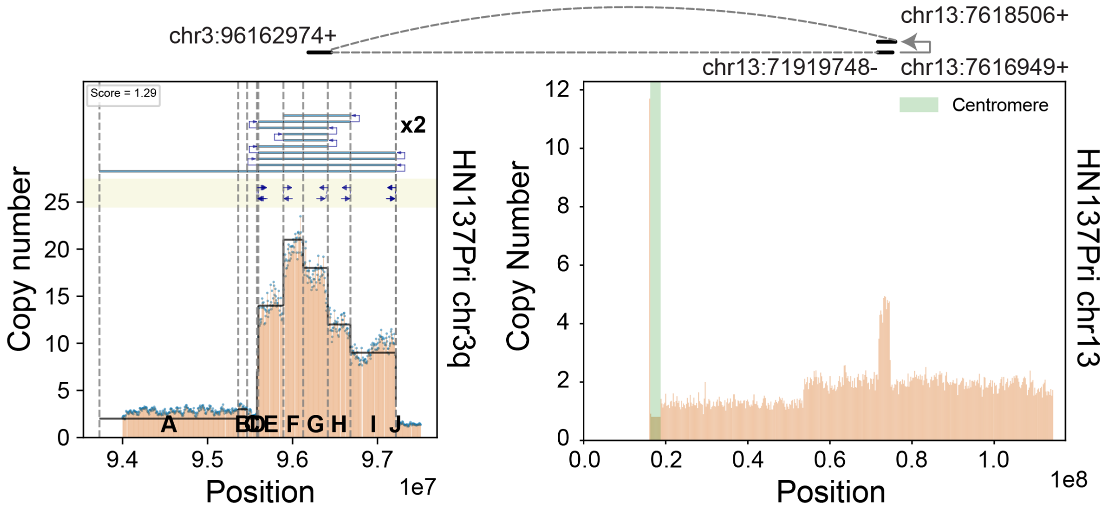

Supplementary Figure S9: A translocation from the BFB region on chr3 to the chr13 in HN137Pri. The translocation and a foldback on chr13 indicate a potential translocation bridge amplification.

Supplementary Methods

S1 ILP formulation

We added a term in the ILP objective function to penalize the case that the resulting BFB sequence contains a foldback, which is not supported by the observed foldback counts. We call the foldback a *missing foldback*. We define binary variables p_k^l and p_k^r to indicate whether missing left and right foldback exist on segment k. For each segment k, $p_k^l = 1$ if and only if the observed left foldback count $L_k = 0$ and the estimated left foldback count $L_k^e > 0$. The same condition applies to right foldbacks. We denote the set of segments with $L_k = 0$ (or $R_k = 0$) as M_k^l (or M_k^r). Thus, the complete objective function is as follows:

$$
\begin{aligned}
\text{minimize} \quad & \sum_{k=1}^{n} \left(\varepsilon_k^c + \varepsilon_k^l + \varepsilon_k^r\right) + \frac{n}{2} \cdot \left(\sum_{k \in M_k^l} p_k^l + \sum_{k \in M_k^r} p_k^r \right) \\
\text{subject to} \quad & \varepsilon_k^c \geq |C_k - C_k^e|, \quad \varepsilon_k^l \geq |L_k - L_k^e|, \quad \varepsilon_k^r \geq |R_k - R_k^e|, \quad \varepsilon_k^c, \varepsilon_k^l, \varepsilon_k^r \geq 0 \\
& p_k^l \leq L_k^e, \quad T \cdot p_k^l \geq L_k^e \quad p_k^r \leq R_k^e, \quad T \cdot p_k^r \geq R_k^e \quad \forall k = 1, 2, \ldots, n.
\end{aligned}
$$

To formulate the ILP constraints in Section 2.3, we define the following symbols:

- Each consecutive sequence is denoted by a tuple of three variables (i, j, d), where $1 \leq i \leq j \leq n$ and $d \in \{+, -\}$
- The set of all consecutive sequence $S = \{(i, j, d) | \forall i, j \text{ s.t. } 1 \leq i \leq j \leq n \text{ and } \forall d \in \{+, -\}\}$
- For any $s \in S$, $\bar{s} \in S$ denotes the reverse complement of s, i.e., they have same i, j but opposite directions d and $\bar{d}$.
- For any $s \in S$, $s' \in S$ denotes a super consecutive sequence of s, i.e., $s' = (i, k, -)$, $\forall k \geq j$ if $s = (i, j, +)$, and $s' = (k, j, +)$, $\forall k \leq i$ if $s = (i, j, -)$.
- Time step $t = 1, \ldots, T$

We also introduce the following binary variables:

- For any $s \in S$, $c_s[t] \in \{0, 1\}$ indicates if the consecutive sequence s is added at time t.
- $p_{t_1,t_2} \in \{0, 1\}$ indicates if the consecutive sequences added between (t_1, t_2) (excluding both endpoints) form a palindrome.
- $m_{s,t',t} \in \{0, 1\}$ indicates if two consecutive sequences added at $t' + 1$ and $t - 1$ are s and $\bar{s}$ (reverse complement of s).
- $v_{s,t',t} \in \{0, 1\}$ indicates if two consecutive sequences added at t' and t are s' (super consecutive sequence of s) and s and $p_{t',t} = 1$.

The ILP constraints can be formulated as follows:

1. For the initial sequence $s_0 \in S$, $c_{s_0}[1] = 1$, where $s_0 = (1, n, +)$ if the BFB region is on the q-arm; otherwise, $s_0 = (1, n, -)$.
2. Empty sequence is palindromic, i.e., $p_{t,t+1} = 1$, $\forall t = 1, \ldots, T - 1$.
3. At each time step t, exactly one consecutive sequence is added, i.e., $\sum_{s \in S} c_s[t] = 1$, $\forall t = 1, \ldots, T$.
4. The directions of adjacent consecutive sequences alternate. Let $w[s] = 1$ if s has direction $+$, and $w[s] = -1$ if s has direction $-$. Then $\sum_{s \in S} w[s] \cdot c_s[t] + \sum_{s \in S} w[s] \cdot c_s[t + 1] = 0$, $\forall t = 1, \ldots, T - 1$.

5. $m_{s,t',t} = 1$ if and only if two consecutive sequences added at t' and t are $\bar{s}$ and s, i.e.,

$$m_{s,t',t} = c_s[t'+1] \wedge c_{\bar{s}}[t-1] \Leftrightarrow \begin{array}{l} m_{s,t',t} \leq c_s[t'+1] \\ m_{s,t',t} \leq c_{\bar{s}}[t-1] \\ m_{s,t',t} \geq c_s[t'+1] + c_{\bar{s}}[t-1] - 1. \end{array}$$

   We then have the match indicator $M_{t',t} = \sum_{s \in S} m_{s,t',t}$ to indicate whether the consecutive sequences added at $t'+1$ and $t-1$ are complementary.
6. For any t', t s.t. $t'+2 \leq t$ and $t-t'-1$ is even, $p_{t',t} = 1$ if and only if he consecutive sequences added between (t', t) (excluding both endpoints) form a palindrome, i.e.,

$$p_{t',t} = p_{t'+1,t-1} \wedge (M_{t',t}) \Leftrightarrow \begin{array}{l} p_{t',t} \leq p_{t'+1,t-1} \\ p_{t',t} \leq M_{t',t} \\ p_{t',t} \geq p_{t'+1,t-1} + M_{t',t} - 1. \end{array}$$

   Note that $p_{t',t} = 0$ for any t', t s.t. $t'+2 \leq t$ and $t-t'-1$ is odd.
7. For any t' and t ($t' < t$), $v_{s,t',t} = 1$ if and only if two consecutive sequences added at t' and t are s' (super consecutive sequence of s) and s, and $p_{t',t} = 1$, i.e.,

$$v_{s,t',t} = c_{s'}[t'] \wedge p_{t',t} \Leftrightarrow \begin{array}{l} v_{s,t',t} \leq \sum_{s' \in S} c_{s'}[t'] \\ v_{s,t',t} \leq p_{t',t} \\ v_{s,t',t} \geq \sum_{s' \in S} c_{s'}[t'] + p_{t',t} - 1. \end{array}$$

8. For any consecutive sequence s added at time point t there must be at least one super consecutive sequence s added at a previous time point t', i.e., $c_s[t] \leq \sum_{t'<t} v_{s,t',t}$, $\forall t = 2, 3, \ldots, T$.

S2 BFB simulation

We developed a BFB-simulation tool with the following steps: (a) generate a canonical BFB sequence as a sequence of prefix inverted duplications of genomic segments; (b) assign lengths and genomic coordinates; (c) simulate reads for a specific technology; and, (e) combine these reads with reads from a normal genome to generate the BFB(+) sample. These steps are elucidated below.

Canonical BFB sequence. We simulate a BFB sequence by modeling a telomeric break followed by a series of prefix inverted duplications. Starting with a BFB template $12\ldots n$, we simulate the first BFB cycle by randomly selecting a suffix $i \ldots n$, where $1 < i \leq n$, reversing the suffix (inverted duplication), and appending it to the BFB template. After the first BFB cycle, we have a new BFB sequence $12\ldots n\overline{n(n-1)\ldots i}$. We then iteratively extend the BFB sequence by reversing and appending a randomly chosen suffix until the BFB sequence exceeds a specified length in terms of the number of segments.

BFB nucleotide sequence. Using a reference genome and a genomic region as input, we generate genomic coordinates for the n segments by randomly partitioning the genomic region into n continuous segments, where each segment has a length of at least 500 kb. Next, we also simulate gaps (10 - 2,000 bp) between breakpoints of foldback inversions in the BFB sequence. For example, if there is a foldback between segments i and $\bar{i}$, we delete the first 10 - 2,000 bp of the 5' end of segment $\bar{i}$. Given a randomly chosen segment with length l, we also simulate a deletion with a length randomly chosen from $0.25l$ to $0.5l$. Note that a deletion in segment i is inherited by all following segments i generated in the BFB

process. With the simulated BFB sequence and genomic coordinates, we generate a FASTA file from the reference genome that contains the nucleotide sequence of the simulated BFB sample.

ONT reads. We then run NanoSim (Yang et al., 2017) (v3.1.0) on the FASTA file with a model from ecSimulator (Zhu et al., 2024) (version 0.7.1, https://github.com/AmpliconSuite/ecSimulator) to sample ONT reads from the BFB sequence with an average coverage of 15×. The sequencing error rate is 3.79%, and the mean read length is 5267 bp. We also simulate ONT reads from normal whole-genome samples using NanoSim with the same parameters. We combine the BFB reads and normal reads at the whole-genome scale for SV calling and CN estimation. As a result, we can simulate a BFB sequence with the corresponding ONT sequencing data.

BFB(-) samples. To simulate BFB(-) samples, we use ecSimulator with default parameters. We first use the module for focal amplification simulation to generate an ecDNA sequence with at least two foldback inversions by customizing the SV frequency in the configuration file. We also generate the sequence of tandem duplications by concatenating a series of sequences with foldbacks. We then run the module for Nanopore read simulation to sample ONT reads from the simulated sequence and generate ONT sequencing data with the same parameters for simulating BFB(+) samples. We also combine the ecDNA (or tandem duplication) reads and normal reads at the whole-genome scale for SV calling and CN estimation.
